# Hypoxic-Ischemic Encephalopathy: Impact on Retinal Neurovascular Integrity and Function

**DOI:** 10.18502/jovr.v16i3.9427

**Published:** 2021-07-29

**Authors:** Ismail S. Zaitoun, Nader Sheibani

**Affiliations:** ^1^Department of Ophthalmology and Visual Sciences, University of Wisconsin School of Medicine and Public Health, Madison, WI, USA; ^2^McPherson Eye Research Institute, University of Wisconsin School of Medicine and Public Health, Madison, WI, USA; ^3^Department of Cell and Regenerative Biology, University of Wisconsin School of Medicine and Public Health, Madison, WI, USA; ^4^Department of Biomedical Engineering, University of Wisconsin School of Medicine and Public Health, Madison, WI, USA

Hypoxic-ischemic encephalopathy (HIE) along with its impact on vision has been recognized for some time. HIE, one of the most common brain injuries, results from secondary oxygen deprivation and blood flow reduction to the brain. Its incidence ranges from 1 to 8 per 1,000 live births in developed countries.^[1]^ A considerable proportion of HIE patients display visual impairment,^[2,3]^ which was considered to be solely due to lesions in the brain neural visual structures and processing.^[4]^ Although recent preclinical studies suggest a direct impact on the retinal visual function, the underlying mechanisms and the retinal cells targeted and affected in response to HIE remain unknown.

The preclinical Rice-Vannucci model is a frequently used acute HIE model in mouse and rat pups.^[5,6,7,8,9]^ This hypoxic-ischemic model is attained by constant occlusion of only one of the common carotid arteries (CCA) followed by exposure of the animal to an air mixture with low oxygen. Exposure to these conditions results in brain damage specific to the hemisphere on the side of the ligated CCA.^[10]^ The CCA provides blood supply to the ophthalmic artery that renders the eye susceptible to HIE insult. This model is frequently used to study the effect of HIE on structural and functional integrity of the brain. In addition, this model has been used to demonstrate the damaging effect of the HIE on the blood vessels^[11]^ or the neurons^[12,13]^ of the rat retina. The development and homeostasis of both the neuronal and the vascular systems are interconnected.^[14]^

To the best of our knowledge, we were first to report a detailed characterization of neurovascular damage in the neonatal mouse retina after exposure to Rice-Vannucci HIE model.^[15]^ We examined the effect of HI exposure of postnatal day 9 (P9) neonatal mice on retinal neurovascular integrity.^[15]^ The mouse retina, like in the human, has three layers of blood vessels. Our studies showed that blood vessels in different layers were either degenerated or failed to form. Similarly, our studies demonstrated that exposure to HI conditions induce neuronal degeneration and glial activation. Observed vascular and neuronal damages were irreversible and most noticeable in the periphery of the retina.

Ischemic stroke can also occur in juvenile humans and result in devastating and lasting disabilities including vision loss. We recently reported on the effect of HI exposure of juvenile mice (age 30 days) on the functional and structural integrity of the retinal blood vessels and neurons.^[16]^ These studies revealed that blood vessels were damaged and that structural and functional integrity of the neurons of the injured retinas were affected as the b-wave and the inner retinal layers were compromised.

Of note, we found retinal damage after exposure to HI vary among individuals; some show mild to no damage, some show moderate injuries, and others show severe injuries. Similar interindividual variabilities in the degree of damage after HIE exposure were reported in mouse, rat, and human neonatal brains^[7,17,18,19]^ and retina.^[13,20,21,22]^

It is unknown why different individuals respond differently after exposure to the same HI conditions. Maturation status of blood vessels and neurons could contribute to the severity of the damage after exposure to ischemic stroke. This is in line with the fact that we found the occurrence of severe retinal vascular damage to be much higher in neonates as compared with juvenile mice. Also, the magnitude of the severe damage was far more apparent and devastating in the neonate mice. Thus, age seems to play a significant role. It is also possible that variation in individual's hemodynamic properties could contribute to the HI-caused damage,^[23]^ since the vascular structure and hence hemodynamic properties varies among retinas. Also, retinas of male and female animals may show different severity of injuries after exposure to the same HI conditions, as in the brain.^[24,25]^

Recent clinical studies have also demonstrated that HIE results in direct retinal damage in human neonates. At least 24.3% of eyes of newborns with a history of HIE were found to have retinal hemorrhage.^[22,26]^ An independent study examined the retinal integrity of two infants diagnosed with HIE using a handheld spectral domain optical coherence tomography (SD-OCT) imaging system. One infant with a history of moderate HIE was found to have severe retinal damage as manifested by thinning of all layers of the retina. The second infant with a history of severe HIE had subretinal fluid but without other obvious retinal damage. These findings support the notion that the retina is a prime target of HI insult, and the severity of retinal damage is independent of the severity of brain damage. A more recent report found three out of eight (37.5%) examined infants with HIE to have various types of retinal damage, such as macular cystoid spaces, thinning in the retinal ganglion cell layer, acute middle maculopathy, and abnormally thin fovea.^[20]^

Preclinical studies including ours and recent human reports demonstrate that both neurons and blood vessels of the retina in neonates and juveniles are vulnerable to HI exposure. These findings call for more clinical attention in assessing the structural and functional integrity of retina of neonates and juvenile humans after exposure to hypoxic-ischemic insults or ischemic stroke. In addition, it is important to start effort in developing interventional methods to prevent and treat HI-induced and ischemic stroke-induced retinal neurovascular damage. The underlying mechanisms that lead to damage of neurons and blood vessels in the retina after exposure to HI conditions remain unknown.

The activation of cell death in brain begins within the first 3–24 hr after exposure to HI conditions.^[27]^ It is likely that critical factors that are responsible for the neurovascular degeneration will be differentially expressed in the early stages of the response to the HI insult. Single cell-RNA sequencing (scRNA-Seq) method can be utilized to determine changes in the transcriptome profiles in the retina after the onset of the HI insult. Identification of altered genes/pathways known to be important for cell death, angiogenesis, and/or neurogenesis could provide more details regarding the underlying mechanisms. These pathways can be targeted for development of new therapeutics to preserve neurovascular functions not only in the brain but also in the retina.

##  Financial Support and Sponsorship

This work was supported in part by an unrestricted award from Research to Prevent Blindness to the Department of Ophthalmology and Visual Sciences; the Retina Research Foundation; NIH grants P30 EY016665, P30 CA014520, S10 OD018221, and EY026078. NS is a recipient of RPB Stein Innovation Award.

##  Conflicts of Interest

There is no conflict of interest.
